# Microwave-assisted catalytic conversion of chitin to 5-hydroxymethylfurfural using polyoxometalate as catalyst

**DOI:** 10.1039/d1ra08560c

**Published:** 2021-12-21

**Authors:** Md. Saidul Islam, Manami Nakamura, Nurun Nahar Rabin, Mohammad Atiqur Rahman, Masahiro Fukuda, Yoshihiro Sekine, Jorge N. Beltramini, Yang Kim, Shinya Hayami

**Affiliations:** Department of Chemistry, Graduate School of Science and Technology, Kumamoto University 2-39-1 Kurokami Chuo-ku Kumamoto 860-8555 Japan hayami@kumamoto-u.ac.jp; Institute of Industrial Nanomaterials (IINa), Kumamoto University 2-39-1 Kurokami Chuo-ku Kumamoto 860-8555 Japan; Priority Organization for Innovation and Excellence, Kumamoto University 2-39-1 Kurokami Chuo-ku Kumamoto 860-8555 Japan; Centre for Tropical Crops and Bio-Commodities, Queensland University of Technology Brisbane 4000 Australia; International Research Center for Agricultural and Environmental Biology (IRCAEB)2-39-1 Kurokami Chuo-ku Kumamoto 860-8555 Japan

## Abstract

The key challenges for converting chitin to 5-hydroxymethylfurfural (5-HMF) include the low 5-HMF yield. Moreover, the disadvantages of traditional acid–base catalysts including complex post-treatment processes, the production of by-products, and severe equipment corrosion also largely limit the large-scale conversion of chitin to 5-HMF. In this view, herein we have demonstrated a microwave aided efficient and green conversion of chitin to 5-HMF while using polyoxometalate (POM) as a catalyst and DMSO/water as solvent. Chitin treated with H_2_SO_4_ followed by ball-milling (chitin-H_2_SO_4_-BM) was selected as the starting compound for the conversion process. Four different POMs including H_3_[PW_12_O_40_], H_3_[PMo_12_O_40_], H_4_[SiW_12_O_40_] and H_4_[SiMo_12_O_40_] were used as catalysts. Various reaction parameters including reaction temperature, amount of catalyst, mass ratios of water/DMSO and reaction time have been investigated to optimize the 5-HMF conversion. The H_4_[SiW_12_O_40_] catalyst exhibited the highest catalytic performance with 23.1% HMF yield at optimum operating conditions which is the highest among the literature for converting chitin to 5-HMF. Significantly, the disadvantages of the state of the art conversion routes described earlier can be overcome using POM-based catalysts, which makes the process more attractive to meet the ever-increasing energy demands, in addition to helping consume crustacean waste.

## Abstract 2

We have demonstrated an efficient conversion of chitin to 5-HMF using a microwave aided method while using polyoxometalate (POM) as catalyst and DMSO/water as solvent.

## Graphical abstract

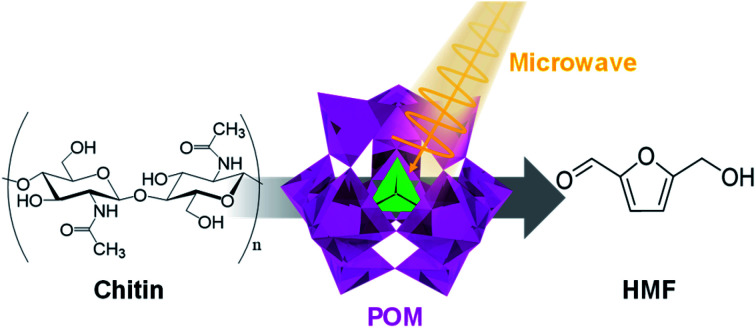

## 1 Introduction

During the past few decades, the conversion of cellulose, which is a polysaccharide of d-glucose and the most abundant biopolymer, into valuable products such as fuels, has been extensively studied as an alternative renewable energy source to meet the increasing energy crisis.^1–4^ On the other hand, chitin biomass which is the second most abundant biopolymer after cellulose and is the main component of the shells of crustaceans such as crabs and shrimps has received less attention primarily because of its intractable molecular structure.^5–9^ Despite the huge annual production and easy availability of chitin-based biomass, it still remains an underutilized resource due to the non-solubility in almost all common solvents which has been identified as a major stumbling block in its appropriate utilization.^5,6,10,11^ Chitin exhibits a very similar chemical structure to that of lignocellulose and consists of a linear polysaccharide composed of *N*-acetylglucosamines (NAG). The basic structural difference between lignocellulose and chitin lies in the functional group at the C2 position, with hydroxyl groups (–OH) in lignocellulose and acetylamino groups (CH_3_CONH–) in chitin. Therefore, the efficient catalytic conversion route from chitin to a value-added platform chemical is being focused on by different research groups worldwide for the proper utilization of chitin-based biomass.

Among the various high-value chemicals, 5-hydroxymethylfurfural (5-HMF) is one of the most promising valuable bio-based platform compounds and is considered to be the key intermediate to bridge the gap between biomass and fossil fuel resources.^12–15^ In a general view, 5-HMF can be used to synthesize many chemicals including furan derivatives and nonfuranic compounds. Furan derivatives, such as 5-alkoxymethylfurfural, 2,5-dimethylfuran, 2,5-furandicarboxylic acid, 2,5-bishydroxymethylfuran, 5-hydroxymethylfuroic acid, and the diether of 5-HMF, have promising potentials in fuel or polymer synthesis. Moreover, nonfuranic compounds, such as adipic acid, caprolactam, caprolactone, levulinic acid, and 1,6-hexanediol, can be converted from 5-HMF.^16^ Therefore, the successful implementation of chitin to 5-HMF conversion technology will lead to a new direction for high-value utilization of chitin biomass in addition to the proper utilization of an enormous volume of waste resources.

In past years, a considerable volume of chitin-based biomass to 5-HMF has been documented.^17,18^ However, most of the previous literature highlighted the 5-HMF conversion using chitosan, a deacetylation product of chitin, due to their benefit of higher solubility in the acidic medium. Nevertheless, the high degree of deacetylation in chitosan for achieving optimum 5-HMF is expensive and associated with rash experimental conditions.^19^ Therefore, the efficient route for direct conversion of chitin to 5-HMF attracted attention in terms of the practical viability of the process. Unfortunately, the use of chitin for direct conversion to 5 HMF is largely limited to their poor solubility and very low yield of product.^20–23^ For example, a very low 5-HMF yield of only 1.9% was reported by Zhang and co-workers using an ionic liquid ([Hbim]Cl) catalyst.^21^ In a separate study, Wang *et al.* observed 5-HMF yield of 9.0% with ZnCl_2_ as a catalyst and at 190 °C for 6 h hydrothermal condition.^22^ The highest conversion efficiency to date of chitin to 5-HMF was achieved as 19.3% using FeCl_2_·4H_2_O as a catalyst by Yu and co-workers.^23^ In addition to the low 5-HMF yield, these processes are limited with critical post-treatment issues. Thus, it is highly desirable for the development of an alternative green catalytic route for improving the efficiency of chitin to 5-HMF conversion.

Polyoxometalates (POMs) are known to be economically viable and environmentally friendly solid catalysts for homogeneous and heterogeneous acid catalyzed reactions.^24,25^ Moreover, the unique physicochemical properties of POMs including strong Brønsted acidity, high proton mobility, and good stability facilitate them as a suitable candidate for biomass conversion. In fact, some previous studies demonstrated the high catalytic activity and environmental friendliness of crystalline cellulose using homogeneous POMs, micellar POMs, and metal salts derivatives of POMs.^26,27^ In addition, the easy recovery of the catalyst after catalytic conversion followed by reuse without decreasing their conversion efficiency has also been reported.^26,27^ Despite a large volume of literature on the production of 5-HMF from glucose and cellulose using POM as a catalyst, the use of POM catalyst for the conversion of 5-HMF from chitin biomass has not been reported yet.

On the other hand, among the physicochemical pre-treatment methods, ball milling is a simple, fast, cost-effective green technology with enormous potential. The ball milling uses friction, collision, shear, or other mechanical actions for the pre-treatment of pristine biomass to modify the crystalline structure.^28^ In particular, the linear structure of the chitin chain forms intra- and intermolecular hydrogen bonds which lead to the chains becoming microfibrils with both crystalline regions and areas of the less ordered or amorphous part. Therefore, efficiently disrupting hydrogen bonding, making many more β-(1→4)-linkages accessible to reactants and catalysts, is critical to increasing the chitin hydrolysis rate. In the current work, we have studied four different POM catalysts including H_3_[PW_12_O_40_], H_3_[PMo_12_O_40_], H_4_[SiW_12_O_40_] and H_4_[SiMo_12_O_40_] for the conversion of chitin to 5-HMF. Moreover, among the chitin conversion products, NAG and levulinic acid (LA) are also measured. The pre-treatment of chitin including acid/base treated followed by ball milling was performed which is believed to decrease the crystallinity and H-bonding network of chitin that facilitates in increasing the solubility of the chitin while maximizing the 5-HMF yield. The H_4_[SiW_12_O_40_] catalyst shows better 5-HMF conversion efficiency over the other three catalysts.

## 2 Experimental

### 2.1. Materials

All reagents are of analytical grade and used without further purification. Four different types of POM catalysts including phosphotungstic acid·30H_2_O (H_3_[PW_12_O_40_]), phosphomolybdic acid·29H_2_O (H_3_[PMo_12_O_40_]), silicotungstic acid·26H_2_O (H_4_[SiW_12_O_40_]), silicomolybdic acid·*n*H_2_O (H_4_[SiMo_12_O_40_]), were purchased from Wako Pure Chemical Industries. Also, Chitin, NAG, LA, acetic acid (AA) and 5-HMF were purchased from Wako Pure Chemical Industries. Formic acid (FA) standard reagent was purchased from Nacalai Tesque Inc.

### 2.2. Ball-milling experiments

The ball-milling (BM) experiments were performed on an ASAHI Rika Manufacturing ball-mill machine. The pure chitin and acid(H_2_SO_4_)/base (NaOH) treated chitin were ball milled to reduce the crystallinity of pristine chitin. The detailed procedures are as follows:

#### Chitin-BM

Chitin (5.0 g) was loaded into a Pot Mill (1.6 L) with an Al_2_O_3_ ball (mass of 1.5 kg and diameter of 1.5 cm). The spinning speed was set at 650 rpm. The milling time was 168 h.

#### H_2_SO_4_ treated chitin-BM

Chitin (5.0 g) was dispersed in 25 mL of diethyl ether containing H_2_SO_4_ (0.28 mL, 0.2 M). The diethyl ether is dried to obtain dry powder. Ball milling experiments were performed in a Pot Mill (1.6 L) with an Al_2_O_3_ ball (mass of 1.5 kg and diameter of 1.5 cm) at a pinning speed 650 rpm for 24 h.

#### NaOH treated chitin-BM

Chitin (5.0 g) and an equivalent amount of NaOH were loaded together into a Pot Mill (1.6 L) with an Al_2_O_3_ ball (mass of 1.5 kg and diameter of 1.5 cm). Ball milling experiments were performed for 24 h. After ball milling, the solid products were collected and washed with methanol repeatedly until pH = 7 followed by oven-dried at 70 °C overnight.

### 2.3. Characterization

The POM catalysts were characterized using FTIR analysis. The powder X-ray diffraction (PXRD) was conducted to observe the change of chitin crystallinity due to ball milling and acid/base treatment. A Rigaku MiniFlex II ultra (30 kV/15 mA) X-ray diffractometer with Cu Kα radiation (*λ* = 1.5406 *λ*) was used to perform PXRD pattern.

### 2.4. Estimation of the solubility of chitin

The solubility of a chitin sample was determined as follows – 500 mg of ball-milled chitin samples (pure and acid/based treated) were added into 20 mL of distilled water. After stirring and sonication for 10 min, the suspension was filtered with a filter paper (no. 5C-40). Thereafter, the undissolved solid was washed, oven-dried, and weighed. The water-soluble product% was calculated as follows:Water-soluble product% = (1 − M_residue/M_feedstock)

### 2.5. Measurement of catalytic activity product analysis

The microwave-assisted conversion of chitin to 5-HMF was carried out using microwave reaction vials. In a typical experimental run, 25 mg of chitin, 5 mL of solvent (H_2_O : DMSO = 1 : 4) and 100 mg catalysts were putted into a 10 mL reactor. After being sealed with a cap, the reactor containing the mixture was mounted in a microwave reactor apparatus (Biotage Initiator+) and heated at a specified reaction time under magnetic stirring. Time zero of the reaction was defined as the time when the reactor reached its set point temperature. Reactions were performed in triplicate to assess the reproducibility of results. The liquids and solid residues were separated by filtration. The liquid sample was collected after reaction and the concentration of the product species were quantified using Agilent Technologies HPLC with a ZORBAX Eclipse Plus C18 as the analytical column and both RID (refractive index) and VWD (UV-Vis) detectors. The HPLC was operated under the following conditions: oven temperature as 35 °C, mobile phase as 5 mM H_2_SO_4_; flow rate of 0.6 mL min^−1^; injection volume of 5 μL. The concentrations of NAG, LA, FA, AA and 5-HMF were quantified through the external standard method and calibration curves of commercially available standard substrates.Yield (%) = ((moles of component in product)/(moles of component in initial reactant)) × 100

## 3 Results and discussion

### 3.1. Characterization of POMs catalyst and Ball-milling effects on chitin crystallinity

**Fig. 1:**
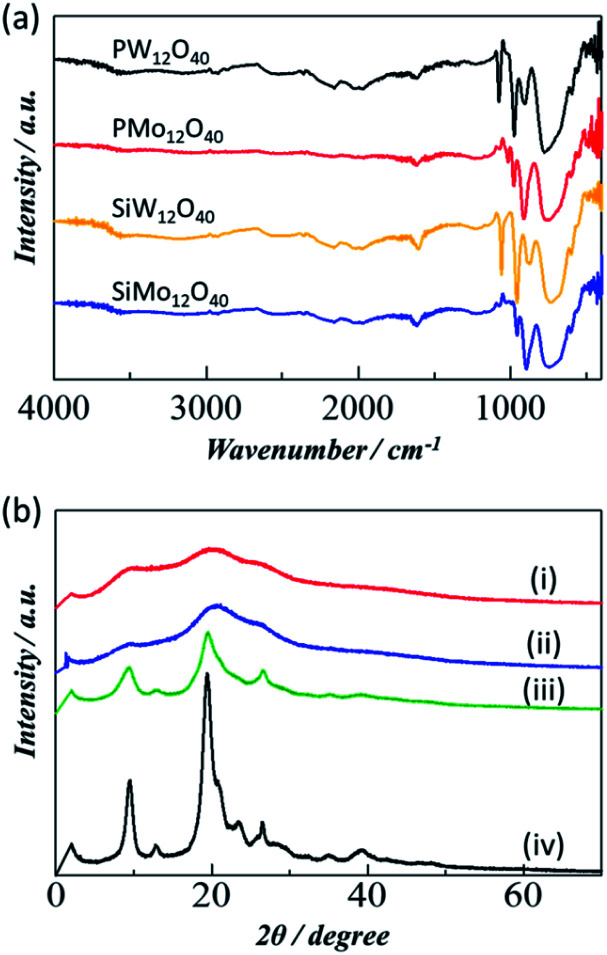
(a) FTIR spectra of different POM catalysts used and (b) PXRD patterns of chitin samples including (i) chitin-BM (ii) chitin-H_2_SO_4_-BM (iii) chitin-NaOH-BM (iv) crystalline chitin.

In the current work, the analytical grade POMs catalysts were purchased from Wako pure chemical company. As we have utilized the POM catalyst as received without further modifications, we have only briefly characterized these catalysts and more detailed information is available on the company website. The characteristic FTIR peaks between 500–1200 cm^−1^ in Fig. 1a confirm the corresponding POM catalyst in their pure form.^29,30^ On the other hand, the chitin crystallinity has been remarkably decreasing after ball milling. In particular, the mechanical forces imported by the ball mill act to alter the crystalline structure of chitin to increase the ratio of amorphous structure which is more reactive and has less tensile strength, and greatly improves the efficiency of hydrolysis. The decrease in crystallinity of the chitin can be observed in the PXRD analysis. The PXRD patterns in Fig. 1b compares the crystallinity behaviour of different chitin samples including pristine crystalline chitin, chitin-BM, chitin-NaOH-BM and chitin-H_2_SO_4_-BM. Clearly, the chitin sample without ball milling exhibited strong peaks at 2*θ* values of 9.4, 19.4, and 23.3°, which were ascribed to the crystalline structure of α-chitin.^31^ In sharp contrast, the PXRD pattern of chitin-BM and chitin-H_2_SO_4_-BM showed broad peaks, indicating that the crystallinity of the chitin had been reduced by the physical pulverization. However, chitin-NaOH-BM did not show much decrease in crystallinity compared to chitin-BM and chitin-H_2_SO_4_-BM.

### 3.2. The solubility and microwave-assisted hydrolysis of different chitin samples

**Fig. 2:**
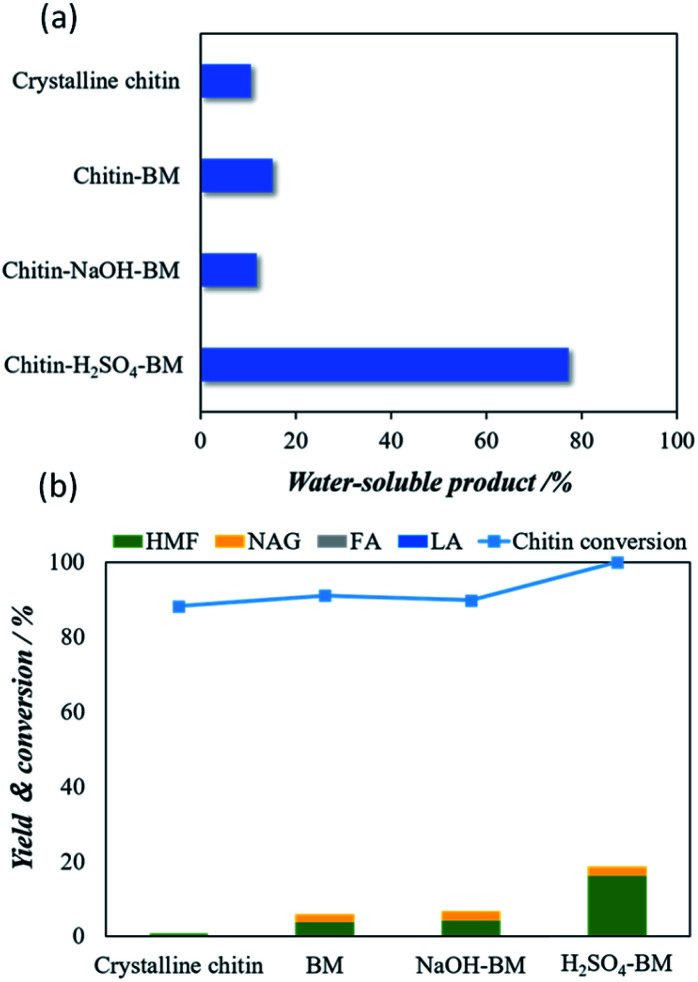
(a) Yields of water-soluble products with different chitin samples and (b) microwave aided Hydrolysis of different chitin samples for converting 5-HMF and other products using H_3_[PW_12_O_40_] catalysts. The hydrolysis reaction was carried out under microwave reaction using 100 mg of H_3_[PW_12_O_40_] as catalyst, 25 mg of chitin, 5 mL of solvent (H_2_O : DMSO = 1 : 4) at 190 °C for 5 min.

Fig. 2a shows the water solubility of pristine crystalline chitin, chitin-BM, chitin-NaOH-BM and chitin-H_2_SO_4_-BM. Although a decrease in crystallinity was observed in all samples after ball milling, the solubility shows the order of chitin-H_2_SO_4_-BM > chitin-BM > chitin-NaOH-BM > Crystalline chitin. Fig. 2b shows the yields of 5-HMF upon hydrolysis of crystalline chitin, chitin-BM, chitin-NaOH-BM and chitin-H_2_SO_4_-BM. The chitin conversion rate of crystalline chitin, chitin-BM and chitin-NaOH-BM chitin samples was about 90% or more. However, the yields of NAG and HMF were low, suggesting that they were converted to water-soluble oligomers. On the other hand, the conversion rate for chitin-H_2_SO_4_-BM is almost 100% while showing a maximum 5-HMF yield of 16.0%. These differences in water solubility and the hydrolysis performance can be attributed to the effect of the acid or base (during ball milling) on the hydrogen bonding amongst the chitin particles. In particular, the use of the catalytic amounts of H_2_SO_4_ in the ball milling process undergoes ‘deep’ depolymerization, while they are predominantly converted into ‘water-soluble’ products. This might lead to a complex mixture of chitin monomers that are more reactive than chitin itself.^32^ Chitin-H_2_SO_4_-BM is selected as starting materials for chitin conversion reaction for the following experiments as it shows the best yield of 5-HMF compared to the other three chitin samples.

### 3.3. Microwave-assisted hydrolysis of chitin-H_2_SO_4_-BM over POM clusters

**Fig. 3:**
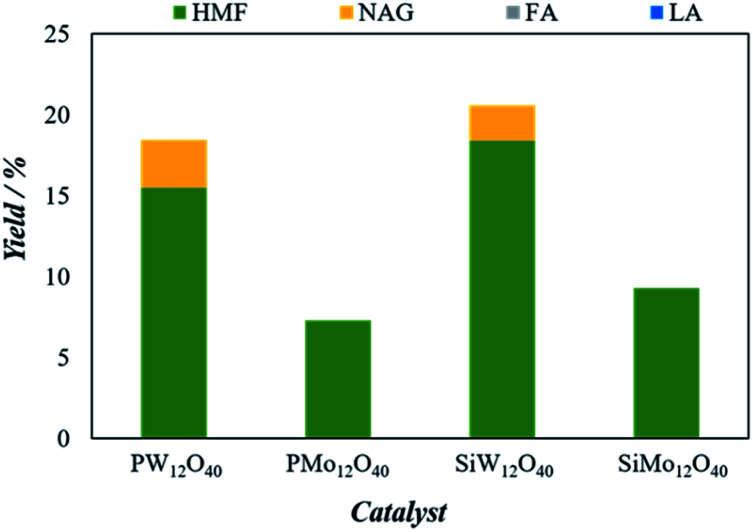
The effects of different types of POM clusters on the hydrolysis of chitin-H_2_SO_4_-BM over H_3_[PW_12_O_40_] (0.035 mmol), H_3_[PMo_12_O_40_] (0.054 mmol), H_4_[SiW_12_O_40_] (0.030 mmol) and H_4_[SiMo_12_O_40_] (0.056 mmol). The reaction was performed with 25 mg of chitin, 100 mg of catalyst, 5 mL of solvent (H_2_O : DMSO = 1 : 4) at 190 °C for 5 minutes.

To justify the catalytic performance of H_3_[PW_12_O_40_], H_3_[PMo_12_O_40_], H_4_[SiW_12_O_40_] and H_4_[SiMo_12_O_40_] catalysts towards the conversion of 5-HMF from chitin, identical microwave reaction conditions were selected. The conversion results of the chitin to 5-HMF and other products are shown in Fig. 3.

Clearly, the 5-HMF yield shows the following order: H_4_[SiW_12_O_40_] > H_3_[PW_12_O_40_] > H_4_[SiMo_12_O_40_] > H_3_[PMo_12_O_40_]. Previously, Izumi *et al.*, characterized the order of softness of heteropolyanions in aqueous solution was estimated as follows: SiW_12_O_40_^4−^ > GeW_12_O_40_^4−^ > PW_12_O_40_^3−^ > PMo_12_O_40_^3−^ > SiMo_12_O_40_^4−^ > NO_3_^−^ > TsO^−^ > SO_4_^2−^ while Keggin anions was marked with having the best softness.^33^ The softness of heteropolyanions is assumed to play an important role in stabilizing organic intermediates. Therefore, the highest yield of 5-HMF was observed with H_4_[SiW_12_O_40_] catalyst effectively hydrolyzed the ball milling chitin up to 18.3% in 5 minutes.

### 3.4. Optimization of reaction parameters

To determine the maximum conversion of 5-HMF from chitin, we have considered H_4_[SiW_12_O_40_] catalyst for further tuning the experimental conditions due to their superior 5-HMF conversion performance over the other three POMs. Several reaction parameters including, reaction temperature, catalyst to feedstock ratio, effect of solvent, and reaction time were optimized to find the best reaction conditions for the conversion of chitin to 5-HMF.

#### 3.4.1. Effect of reaction temperature

**Fig. 4:**
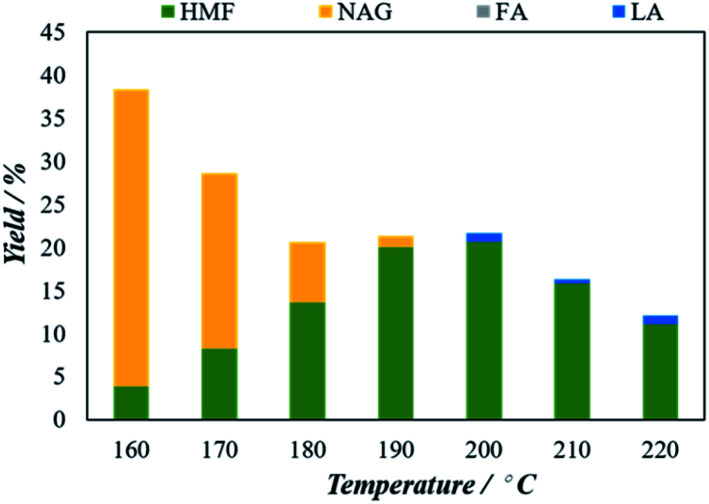
Effect of reaction temperature on the microwave aided chitin conversion using H_4_[SiW_12_O_40_] as catalysts. The reaction was performed with 25 mg of chitin, 100 mg of catalyst, 5 mL of solvent (H_2_O : DMSO = 1 : 4) for 5 minutes.

The effect of reaction temperature on the conversion of 5-HMF from chitin was evaluated while the temperature was varied from 160 °C to 220 °C. The result in Fig. 4 implies that the temperature has a significant impact on the chitin conversion. In particular, the 5-HMF yield gradually increased from 3.8% at 160 °C to 20.5% at 200 °C. Upon further increase the temperature, the 5-HMF yield start to decrease and the value of 16.1 and 13.7% were obtained at 210 °C and 220 °C, respectively. The decrease in 5-HMF yield after increasing reaction temperature beyond 200 °C might be attributed to the fact that 5-HMF is more reactive at higher temperatures and start to dissociate in the reaction media. Interestingly, a considerable amount of NAG was observed at 160 °C. When the temperature is increased, the amount of NAG decreases and almost no NAG is detected beyond 200 °C. This observation might be attributed to the decomposition of NAG at higher temperatures. Based on these results, an optimized microwave condition for the conversion of 5-HMF is 200 °C. In these optimized conditions, the effect of catalyst amount for chitin to 5-HMF conversion was also studied.

#### 3.4.2. Effect of catalyst to feedstock mass ratio

**Fig. 5:**
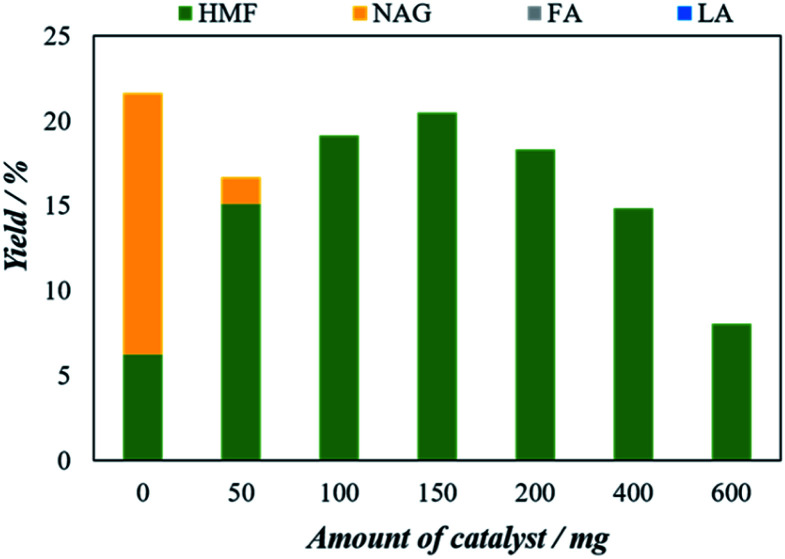
Effect of initial catalyst amount on the microwave aided chitin conversion using H_4_[SiW_12_O_40_] as catalysts. The reaction was performed with 25 mg of chitin, 5 mL of solvent (H_2_O : DMSO = 1 : 4) at 200 °C for 5 minutes.

It is expected that the conversion of chitin to 5-HMF will increase with increasing the catalyst amount. On the other hand, a large amount of catalyst may not only promote the conversion of chitin to 5-HMF but also promote other side reactions such as rehydration of HMF to LA and cross-linking polymerization reactions, which may reduce the 5-HMF yield. Therefore, the suitable amount of catalyst has justified to optimize the chitin to 5-HMF. Fig. 5 represents the effect of the initial amount of catalyst on the 5-HMF yield.

The catalysts amount was varied from 50 mg to 600 mg. The 5-HMF yield of 6.2% in the blank experiment increased steadily with increasing catalyst amount and reaches a maximum of 20.5% of 5-HMF with 150 mg of catalyst. However, when the amount of catalyst was increased the 5-HMF start to decrease. At 200 mg of catalyst, the yield decreases to 18.3% while with 600 mg catalyst the 5-HMF significantly decreased to 8.3%. The results might be attributed to the formation of side reactions (including humins) in the reactive aqueous phase reduced the yield of 5-HMF as the amount of catalyst increased.^20^ The catalyst to feedstock ratio of the optimum 5-HMF yield is 6 : 1. Although the high catalyst to feedstock ratio might lead the whole process uneconomical, however, a much higher catalyst to feedstock ratio (as high as 20 : 1) is reported in most of the previous chitin-based biomass to 5-HMF conversion literature.^22,23^ This high catalyst to feedstock ratio might be attributed to the large number of active sites provided by the catalyst is required for the conversion of chitin-based biomass to 5-HMF which in turn increases the catalyst to feedstock ratio.

#### 3.4.3. Effect of solvent (different mass ratios of water/dimethyl sulfoxide on chitin conversion into 5-HMF)

**Fig. 6:**
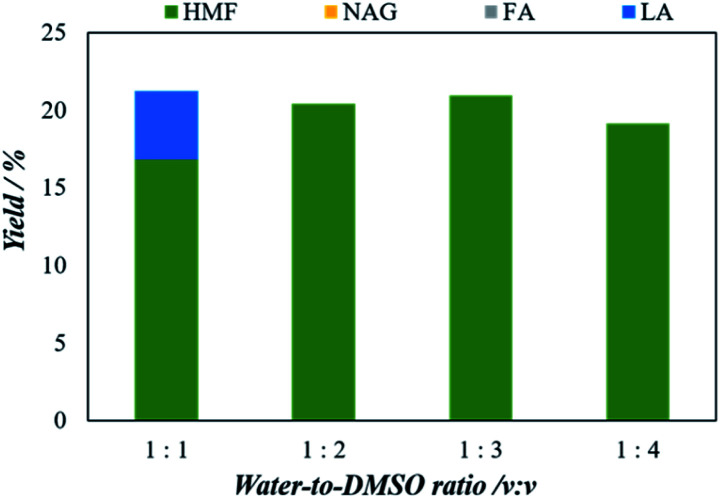
The effect of solvent (H_2_O/DMSO ratio) on the microwave aided chitin conversion using H_4_[SiW_12_O_40_] as catalysts. The reaction was performed with 25 mg of chitin, 100 mg of catalyst, at 200 °C for 5 minutes.

In order to suppress side reactions during catalytic conversion of biomass, dimethyl sulfoxide (DMSO) is widely used as additives with aquas phase.^23,34^ In particular, the dipolar aprotic solvent breaks the intermolecular hydrogen bonds between water and the presence of both water and DMSO pushed the reaction towards 5-HMF formation and inhibited undesirable side reactions.^35,36^ The effect of solvent (H_2_O/DMSO ratio) for chitin to 5-HMF conversion using H_4_[SiW_12_O_40_] (100 mg) at 200 °C for 5 min and are shown in Fig. 6. As the mass ratios of water/DMSO increases from 1 : 1 to 1 : 3, the yield of 5-HMF increases from 16.8% to 21.0%. On the other hand, when the ratio increased to 1 : 4, the yield of 5-HMF decreased. The decrease in the 5-HMF yield for 1 : 4 of H_2_O/DMSO solution may be due to the decrease in the rate of transfer from the aqueous phase of the reaction to the organic solvent, which promoted the simultaneous formation of intermediate compounds and other reactive intermediates by condensation and polymerization of 5-HMF in the aqueous phase.

#### 3.4.4. Effect of reaction time

**Fig. 7:**
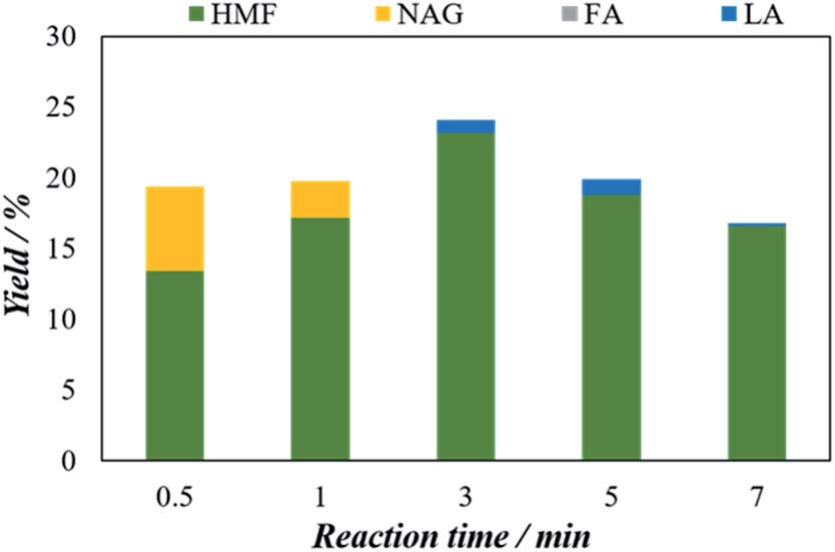
Effect of reaction time on the microwave aided chitin conversion using H_4_[SiW_12_O_40_] as catalysts. The reaction was performed with 25 mg of chitin, 150 mg of catalyst, 5 mL of solvent (H_2_O : DMSO = 1 : 3) at 200 °C.

The effect of reaction time for converting chitin to 5-HMF is shown in Fig. 7 while the reaction time varied from 0.5 min to 7 min. Clearly, chitin to 5-HMF yield increases almost linearly at the initial stage of reaction and increased from 13.8% to 23.1% when the reaction times increased from 0.5 minutes to 3 minutes, respectively.

Then, 5-HMF yield starts to decrease with time and declined to 18.2% at 7 minutes. The 5-HMF yield decreasing after 3 minutes can be attributed to the formation of unwanted by-products principally humins compound *via* condensation, polymerization of 5-HMF and other reactive intermediates in the reaction medium.^37^ Therefore to obtain the optimal 5-HMF yield, the reaction should be set for 3 minutes. Also, as shown in Fig. 7, at the very beginning of the reaction at 0.5 minutes, a certain amount of NAG (6%) has also detected which then disappeared with time which might be converted to the 5-HMF. Thus, the optimal 5-HMF yield is estimated as 23.1% using 3 minutes of reaction time.

### 3.5. Comparison with previously reported chitin to 5-HFM conversion catalytic systems

Among the different chitin-based biomass, chitosan and *N*-acetyl-d-glucosamine are widely studied for 5-HMF conversion and only a few reports are available using pristine chitin which might be ascribed to the low solubility of chitin leading to the lower yield of 5-HMF.^17,18^ In the current work, we have improved the solubility and reduced the crystallinity of chitin using acid treatment followed by ball milling. The microwave-aided process using POM catalyst confirms the comparatively higher 5-HMF yield. Table 1 compares the competitive reported value for the conversion of chitin to 5-HMF.

**Table 1:** Reported values for the conversion of chitin to 5-HMF

Substrate	solvent	Catalyst	Reaction condition	5-HMF yield (%)	Ref.
Chitin	H_2_O	SnCl_4_·5H_2_O	200 °C, 30 min	—	20
Chitin	DMSO/H_2_O	[H_bim_]Cl	180 °C, 30 min	1.9	21
Chitin	67 wt% ZnCl_2_ aqueous solution	ZnCl_2_	120 °C, 6 h	9.0	22
Chitin	40 wt% DMSO–water	FeCl_2_·4H_2_O	190 °C, 6 h	19.3	23
Chitin	67% DMSO–water	H_4_[SiW_12_O_40_]	200 °C, 3 min	23.1	This work

The maximum achieved efficiency of previous literature for conversion of chitin to 5-HMF was 19.3% while in the current we have obtained obvious higher performance of 23.1%. To the best of our knowledge, this is the first report for the conversion of chitin to 5-HMF using POM catalyst while obtaining the highest efficiency among the reported chitin to 5-HMF conversion. The efficient conversion of 5-HMF from chitin in the current work can be attributed to the contribution of (i) ball milling, (ii) microwave aided process and (iii) available Brønsted acidic sites in the POM catalyst. The ball milling with a small amount of H_2_SO_4_ efficiently reduces the crystalline part of chitin and improves the water solubility of the substrate. Microwave can be absorbed deeply into the folding layers of the chitin which improve the contraction between the catalyst and the solid substrate resulting in efficient hydrolysis is achieved. On the other hand, POM catalyst contains both Brønsted acidic and metallic parts in its structure. The Brønsted acidic part makes these compounds behave as strong acid to catalyze the hydrolysis of chitin while the reaction starts with the adsorption of the chitin on the Brønsted sites of the catalyst and protonates the β-1,4-glycosidic bonds to form a positively charged acyclic or cyclic intermediate. Furthermore, the POM catalyst is reported as easily recoverable for reuse without significant loss of their performance.^26,27^ In a typical reuse procedure the catalyst can be recovered from the hydrolytic solution by extraction with diethyl ether after the first catalytic cycle followed by complete evaporation of the diethyl ether. The recovered catalyst is then used for a second run under the same conditions. The reported efficiencies for recyclability of POM catalysts are over 90%.^26,27^ Unfortunately, the cycle useability experiment of POM catalysts is beyond our current experimental scope and will include in our upcoming work.

## 4 Conclusions

The green conversion of chitin-based biomass to a value-added platform chemical holds enormous potential to contribute as a renewable energy source to meet the increasing energy demand in addition to help consuming crustacean waste. In the current study, we conducted microwave-aided chitin biomass to 5-HMF conversion using POM clusters as a catalyst. The reduction in crystallinity and improvement in the solubility of pristine chitin were achieved by sulfuric acid-treated chitin biomass followed by ball milling. Among four different POMs utilized, H_4_[SiW_12_O_40_] showed the best catalytic performance for the conversion of chitin to 5-HMF with a conversion efficiency of 23.1% using the microwave at 200 °C and 150 mg of catalysts for 3 min in 67% DMSO–water solvent. We believe that the results of the current study will provide a basis for an important future guide to the utilization of widely available and second most abundant chitin-based biopolymer to the platform chemicals.

## Conflicts of interest

There are no conflicts to declare.

## Supplementary Material
